# The State Medical Association of Alabama

**Published:** 1877-05-20

**Authors:** 


THE State Medical Association of Alabama, at Birmingham, elected Dr. Peter Bryce, President; Dr. Frank Prince and Dr. Lampley, Vice-Presidents; Dr. B. H. Riggs, Orator.

